# Production of IFN-β during *Listeria monocytogenes* Infection Is Restricted to Monocyte/Macrophage Lineage

**DOI:** 10.1371/journal.pone.0018543

**Published:** 2011-04-11

**Authors:** Evgenia Solodova, Jadwiga Jablonska, Siegfried Weiss, Stefan Lienenklaus

**Affiliations:** Department of Molecular Biotechnology, Helmholtz Centre for Infection Research, Brunswick, Germany; Indian Institute of Science, India

## Abstract

The family of type I interferons (IFN), which consists of several IFN-α and one IFN-β, are produced not only after stimulation by viruses, but also after infection with non-viral pathogens. In the course of bacterial infections, these cytokines could be beneficial or detrimental. IFN-β is the primary member of type I IFN that initiates a cascade of IFN-α production. Here we addressed the question which cells are responsible for IFN-β expression after infection with the intracellular pathogen *Listeria monocytogenes* by using a genetic approach. By means of newly established reporter mice, maximum of IFN-β expression was observed at 24 hours post infection in spleen and, surprisingly, 48 hours post infection in colonized cervical and inguinal lymph nodes. Colonization of lymph nodes was independent of the type I IFN signaling, as well as bacterial dose and strain. Using cell specific reporter function and conditional deletions we could define cells expressing LysM as the major IFN-β producers, with cells formerly defined as Tip-DCs being the highest. Neutrophilic granulocytes, dendritic cells and plasmacytoid dendritic cells did not significantly contribute to type I IFN production.

## Introduction

Interferons (IFN) were first discovered over 50 years ago by Isaacs and Lindenmann [1]. Due to its complexity, the IFN system is still little understood and remains a subject of intensive research. Currently, three types of IFNs are distinguished – type I, type II consisting of only IFN-γ, and the recently discovered type III also called IFN-λ. Here, we will focus on type I IFN. This family of cytokines comprises a single IFN-β, more than 13 IFN-α, and several other less well characterized members [2], [3]. All these subtypes signal through a common cell surface receptor (IFNAR) to activate IFN-inducible genes that exert a wide range of effects central to innate and adaptive immunity [2]. As a rule, IFN-β is produced as the earliest of type I IFN initiating a cascade of IFN-α via autocrine and paracrine loops [4], [5]. In addition, during viral infections type I IFN may be induced in plasmacytoid dendritic cells (pDCs) also named “natural interferon producing cells”. Due to constitutive expression of the transcription factor IRF-7, pDCs are able to immediately produce large amounts of IFN-α [6]. Non-viral pathogens (i.e., bacteria, protozoa, fungi and helminthes) may also induce type I IFN. Interestingly, in contrast to viral infections where IFNs are normally protective, in non-viral infections IFN production might be defensive or deleterious [7], [8].

The prototype of a deleterious type I IFN response during bacterial infection is experimental listeriosis elicited by the gram-positive rod-shaped intracellular bacterium *Listeria monocytogenes* that was discovered in 1926 (reviewed in [9]). *L. monocytogenes* induces type I IFN synthesis via triggering a still uncharacterized pattern recognition receptor in the cytosol of host cells [10], [11]. Cyclic diadenosine monophosphate (c-di-AMP), secreted by *L. monocytogenes*, might be one of the cytosolic activators of type I IFN [12]. In addition, lymphotoxin-α might be involved in the triggering of type I IFN responses [13], [14] and lymphotoxin β receptor was shown to be crucially involved in the controlling of *Listeria monocytogenes* infection [15].

Secretion of type I IFN increases susceptibility to Listeria by inducing apoptosis of T cells and macrophages [16]–[18]. In accordance, mice deficient of IFNAR are more resistant to Listeria and show decreased T cell apoptosis in the spleen during infection [17], [19], [20]. Moreover, type I IFN are able to down regulate IFN-γ receptor thereby suppressing macrophage activation and increasing host susceptibility to Listeria infection [21], [22].

Intravenously inoculated *L. monocytogenes* is taken up by the spleen and removed from the blood predominantly by mononuclear phagocytes in the marginal zone of the white pulp [23]. Myeloid cells, especially monocytes and macrophages are rapidly recruited to sites of bacterial infection and are required for initial control of *L. monocytogenes* infection [11], [24]–[26]. During bacterial infections, circulating monocytes are known to differentiate into tissue macrophages and dendritic cells (DCs) [27]. Additionally, inflammatory Gr1^+^/Ly6C^high^ monocytes are able to differentiate into tumor necrosis factor-α (TNF-α) and inducible nitric oxide synthase (iNOS) producing so-called Tip-DCs at sites of infection [11].

According to recent publications, different myeloid cell populations were shown to be responsible for IFN-β production during Listeria infection. Stockinger et al. defined CD11b^+^CD11c^−^PDCA1^−^B220^−^ macrophages to be the major IFN-β producers and pDCs were claimed to make no contribution [28]. On the other hand, Dresing et al. observed IFN-β exclusively in the myeloid cell population called Tip-DCs [29]. In the present study, we used a genetic approach to address the question which cells produce IFN-β during experimental listeriosis.

Previously, we generated a conditional mouse line, in which the IFN-β coding sequence is replaced by the reporter luciferase upon cell specific Cre expression [30]. Here we explored CD19cre, CD4cre and LysMcre mice to activate the reporter function in B cells, T cells, monocyte/macrophages and neutrophils. As reporter mice, we used mice that were heterozygous for the targeted mutation. This allowed IFN-β production from the functional wild type allele. To demonstrate the physiological relevance of IFN-β produced by the cell types mentioned above, we included mice homozygous for the conditional deletion and compared them with mice in which IFN-β was deleted in germ line. Overview of transgenic mice used in this paper is presented in Figure S1.

Using these genetically modified mice, we highlight the importance of IFN-β during the deleterious action of type I IFN in the course of Listeria infection. In addition, the novel reporter mice revealed a maximum of IFN-β induction 24 hours post infection (p.i.) in spleen and, surprisingly, 48 hours p.i. in cervical and inguinal lymph nodes after high dose intravenous infection. Low dose infection followed the same pattern of IFN-β production, although with delayed kinetics. The luciferase signal in spleen and lymph nodes mirrored the presence of Listeria in these organs. Moreover, our results showed that LysM-expressing cells are the main producers of IFN-β during murine listeriosis, especially the cells previously defined as Tip-DCs. Finally, neutrophils, DCs and pDCs and did not significantly contribute to type I IFN production under our conditions of *L. monocytogenes* infection.

## Results

### During *Listeria monocytogenes* infection, IFN-β is produced in spleen and lymph nodes

**Figure 1:**
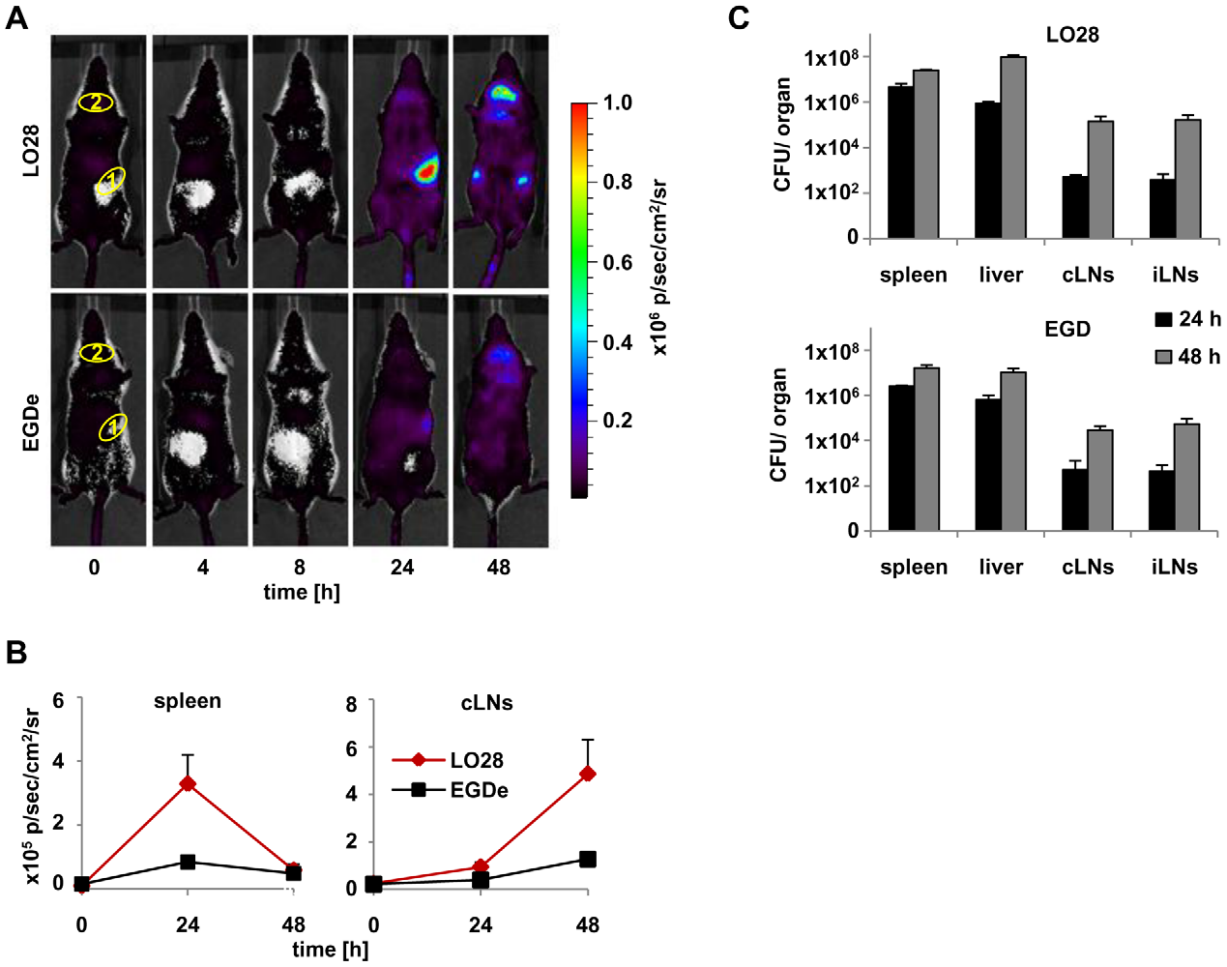
Induction of IFN-β after *L. monocytogenes* infection is restricted to spleen and lymph nodes. **A**) Albino IFN-β^+/Δβ-luc^ mice on the C57BL/6 background were infected intravenously (i.v.) with 5×10^5^ *L. monocytogenes* LO28 and EGDe strains. At the indicated time points, mice were injected with luciferin (i.v.) and luciferase activity was visualized in the IVIS 200 whole body imaging system. The areas encircled in yellow are the regions of interest used for quantification in Fig. 1B. **B**) Quantification of *in vivo* imaging presented in Fig. 1A by measuring of luminescence intensity within the selected regions of interest (1 and 2 for spleen and cervical lymph nodes, respectively) at the depicted time points. **C**) C57BL/6 mice were infected i.v. with 5×10^5^ *L. monocytogenes* LO28 and EGDe strains. Bacterial loads of indicated organs were determined at 24 and 48 hours post infection. White spots observed at the 0/4/8 hour time points represent areas below the set detection limit. “cLNs” and “iLNs” stand for cervical and inguinal lymph nodes respectively. Graphs are taken from 1 representative experiment with 5 mice per group. The experiment was repeated twice.

Different strains of *Listeria monocytogenes* vary in their ability to activate type I IFN production [31]. Here we compared LO28 and EGDe, two commonly used strains of *Listeria monocytogenes*. First, we asked when and where type I IFN is induced. To address this question, we used a previously described IFN-β reporter mouse, which allows whole body *in vivo* imaging of IFN-β induction using firefly luciferase as a reporter [30]. Figure 1A shows induction of IFN-β in albino IFN-β^+/Δβ-luc^ reporter mice after intravenous (i.v.) injection of *Listeria monocytogenes* LO28 and EGDe strains. At 24 hours post infection (p.i.), IFN-β induction occurred almost exclusively in the spleen. Interestingly, 48 hours p.i. a bright luminescent signal appeared in the cervical and in the inguinal lymph nodes, especially when using the LO28 strain. At this time, no production of IFN-β was detectable in the spleen any longer. Loss of the signal from the spleen 48 hours p.i. could be due to complete destruction of spleen together with the massive apoptosis of lymphocytes [32]. Quantitation of luciferase activity in the selected regions of interest (Fig. 1A, circles 1 and 2 for spleen and cervical lymph nodes, respectively) showed that LO28, induced a stronger signal compared to EGDe (Fig. 1B), although colonization by bacteria was comparable (Fig. 1C). This confirmed the findings by Reutterer et al. that LO28 is the most potent type I IFN stimulator amongst the commonly used Listeria strains [31]. Of note, there was no significant signal from the liver at any time point, although this organ is highly colonized by Listeria.

### *Listeria monocytogenes* colonizes cervical and inguinal lymph nodes

Systemic application of *L. monocytogenes* is used in many studies related to host-pathogen interaction [9], [33], [34], and spleen and liver were defined as the target organs of these bacteria [23]. Little attention has been paid to lymph nodes until now. Our finding that IFN-β is produced in the lymph nodes lead us to test whether lymph nodes are also colonized by Listeria after i.v. infection. Indeed, already 24 hours p.i. we could observe bacteria in cervical and inguinal lymph nodes independent of the bacterial strain used (Fig. 1C). Between 24 and 48 hours p.i., bacterial burdens in lymph nodes increased about 100 fold. Colony forming units of Listeria in spleens and livers also increased although not as dramatic.

**Figure 2:**
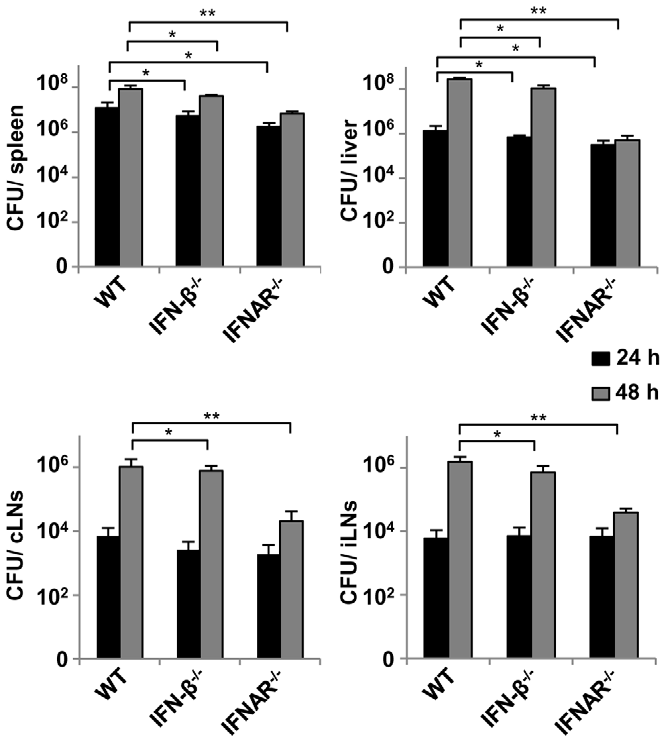
Colonization of spleen, liver, cervical and inguinal lymph nodes is independent of type I IFN. C57BL/6, IFN-β^−/−^ and IFNAR^−/−^ mice were infected intravenously with 5×10^5^ *L. monocytogenes* LO28. Bacterial loads of indicated organs were determined 24 and 48 hours after infection. “cLNs” and “iLNs” stand for cervical and inguinal lymph nodes respectively. Experiment was done twice with 3 animals per group. Results are expressed as means. Student's t-test was used for statistical analysis. *p<0.05, **p<0.001.

To investigate the influence of type I IFN on lymph node colonization, we compared wild type C57BL/6, IFN-β^−/−^ and IFNAR^−/−^ mice. Like in spleen, a defect in the type I IFN system resulted in lower bacterial numbers (Fig. 2) confirming a detrimental role of these cytokines also in lymph nodes. These differences were only detectable at 48 hours p.i., consistent with the peak of IFN-β production at this time point.

**Figure 3:**
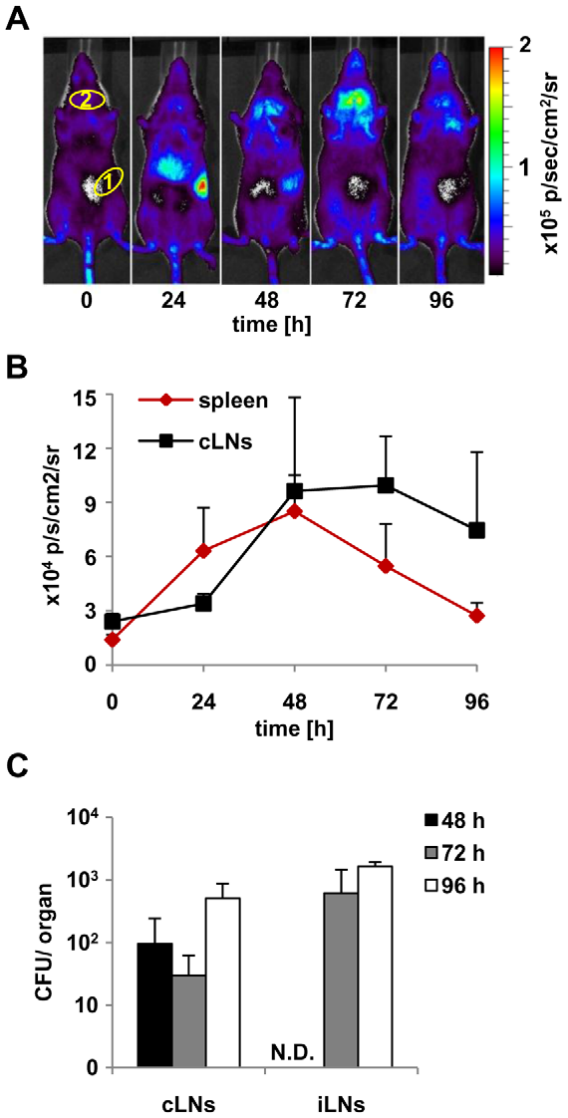
Colonization of and IFN-β production in lymph nodes after low dose infection. Albino IFN-β^+/Δβ-luc^ mice on C57BL/6 background were infected intravenously (i.v.) with 2×10^3^ *L. monocytogenes* LO28. **A**) At the indicated time points, mice were injected with luciferin (i.v.) and luciferase activity was visualized in the IVIS 200 whole body imaging system. The areas encircled in yellow are the regions of interest used for quantification in Fig. 3B. **B**) Quantification of *in vivo* imaging presented in Fig. 3A by measuring of luminescence intensity within the selected regions of interest (1 and 2 for spleen and cervical lymph nodes, respectively) at the depicted time points. **C**) Bacterial loads of organs at different time points post infection. N.D. indicates not detected. “cLNs” and “iLNs” stand for cervical and inguinal lymph nodes, respectively. Graphs are taken from 1 representative experiment with 6–10 mice per group. The experiment was repeated twice.

To rule out whether lymph node colonization is specific and is not due to the high dose of infection, we first monitored albino IFN-β^+/Δβ-luc^ reporter mice for IFN-β production after low dose *Listeria monocytogenes* infection (Fig. 3A). Luciferase expression at 24 hours p.i. peaked in spleen. At 48 and 72 hours p.i. we observed the signal both in spleen and in cervical lymph nodes. At 96 hours p.i. the IFN-β production pattern was almost identical to the 48 hour time point after high dose infection (Fig. 1A), although the overall luminescent signal was lower (note the different scales). Quantification of luminescence intensity in regions of interest (Fig. 3B) in spleen and cervical lymph nodes (Fig. 3A, selected circled areas 1 and 2, respectively) confirmed qualitative *in vivo* data presented in Fig. 3A.

As expected, colonization of lymph nodes paralleled IFN-β production (Fig. 3C). These observations suggested that lymph node colonization by *L. monocytogenes* with subsequent IFN-β production is general phenomenon and does not depend on the bacterial dose, strain or type I IFN signaling.

### pDCs do not contribute to the production of type I IFN during *Listeria monocytogenes* infection

**Figure 4:**
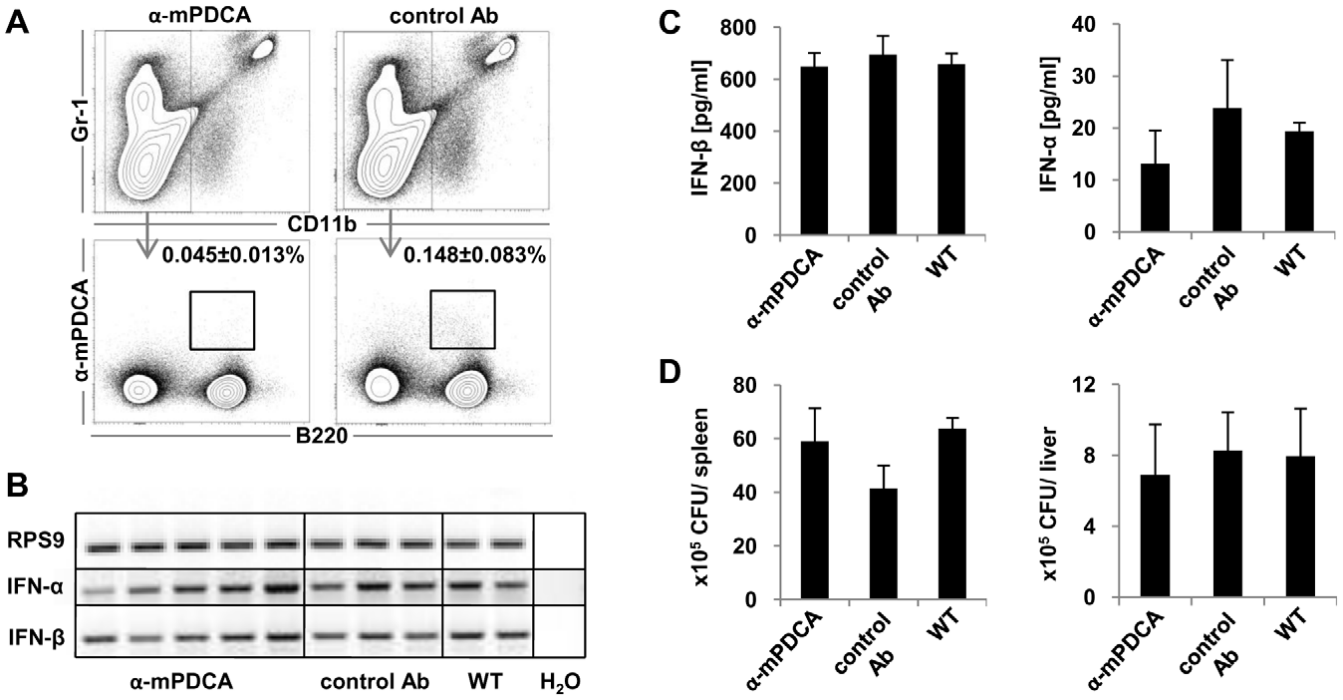
Plasmacytoid dendritic cells do not produce type I IFN during *Listeria monocytogenes* infection. pDCs were depleted *in vivo* by intravenous (i.v.) injection of 100 µg anti-mPDCA-1 mAb 24 hours prior *L. monocytogenes* LO28 infection. As isotype control, rat IgG was used. C57BL/6 mice were infected with 5×10^5^ bacteria i.v., 24 hour post infection spleens and sera were harvested. **A**) Gating strategy and percentage of pDCs in spleens. **B**) Expression of IFN-β and IFN-α was analyzed by RT-PCR. RPS9 was used as housekeeping gene to control the amount of cDNA employed in the assay. **C**) Serum levels of IFN-β and total IFN-α were analyzed by ELISA. **D**) Bacterial loads from spleen and liver. “α-mPDCA” stands for mice depleted of pDCs; “control Ab” stands for mice injected with isotype control IgG; “WT” stands for mice injected with PBS. Graphs display one representative experiment with 3–5 mice per group. The experiment was repeated 3 times.

In response to a wide variety of enveloped DNA and RNA viruses (e.g., Influenza virus, vesicular stomatitis virus) as well as parasites (*Plasmodium falciparum*) and CpG oligonucleotides, IFN type I is produced by pDCs [35]. *L. monocytogenes* was shown after intragastric administration to activate pDCs in spleen and in mesenteric lymph nodes as detected by an increased expression of MHC II and CD86 [36]. By extrapolation, a contribution of pDC to the type I IFN response after systemic Listeria infection could be expected, although *in vitro* stimulation of splenic pDC with Listeria did not induce type I IFN [28]. To evaluate the contribution of pDC to the overall IFN production *in vivo* after *Listeria monocytogenes* infection, we performed depletion experiments. To this end, 24 hours prior to infection mice were intravenously injected with the depleting antibody anti-mPDCA-1. 24 hours p.i. spleens were analyzed for the presence of pDCs. Figure 4A demonstrates gating strategy for pDCs and depletion efficiency.

RT-PCR of spleen cells 24 hours p.i. showed that mRNA expression of IFN-β and total IFN-α were not altered regardless of pDC depletion (Fig. 4B). Similarly, serum levels of these cytokines did not reveal any significant differences between mice depleted of pDCs or control mice (Fig. 4C). Moreover, bacterial loads in spleens and livers of such mice were also similar (Fig. 4C). Hence, we conclude that pDCs do not significantly contribute to type I IFN production and also do not have a physiological relevance with regard to colonization of spleen and liver during murine listeriosis.

### LysM-expressing cells produce IFN-β both in spleen and in lymph nodes

**Figure 5:**
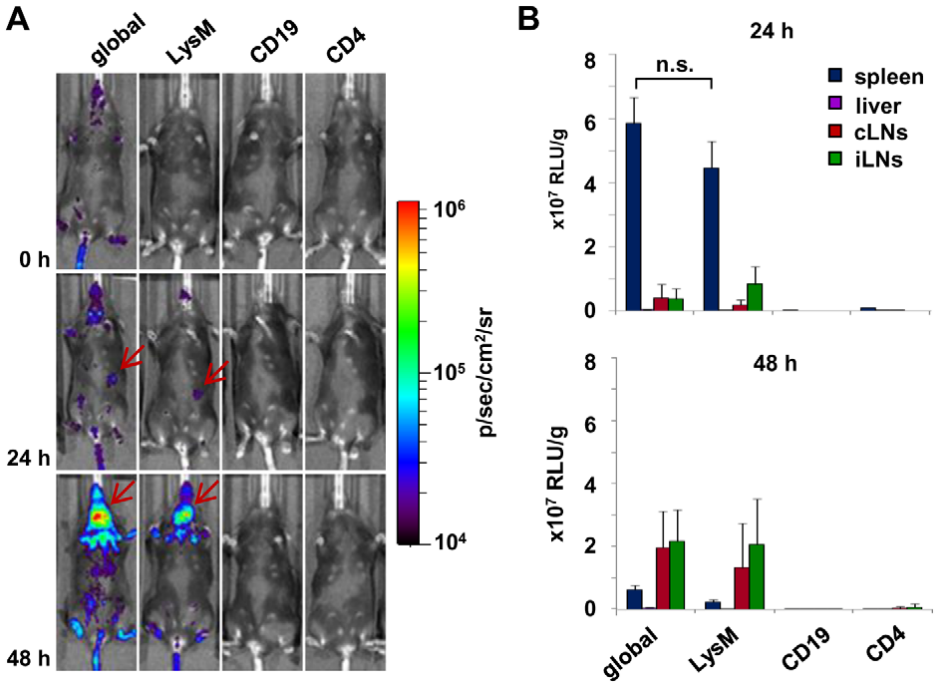
Monocytes/macrophages/neutrophils produce IFN-β in spleen, cervical and inguinal lymph nodes. Mice of indicated phenotypes were infected intravenously (i.v.) with 5×10^5^ *L. monocytogenes* LO28. Mice are on the C57BL/6 genetic background. The albino gene was not yet crossed in. “global” stands for IFN-β^+/Δβ-luc^; “CD4”, “CD19” and “LysM” stand for IFN-β^+/floxβ-luc^ x CD4cre, IFN-β^+/floxβ-luc^ x CD19cre and IFN-β^+/floxβ-luc^ x LysMcre, respectively. **A**) At the depicted time points after infection, mice were injected with luciferin (i.v.) and luciferase activity was visualized in the IVIS 200 imaging system. Low signals are due to quenching of the bioluminescent light by melanin in fur and skin of the C57BL/6 mice. Red arrows indicate location of spleen and cervical lymph nodes at 24 and 48 hours post infection, respectively. **B**) For quantification of luciferase activity, indicated tissues were harvested 24 and 48 hours post infection and organ homogenates were analyzed in a luminometer. “cLNs” and “iLNs” stand for cervical and inguinal lymph nodes respectively. Experiment was done twice with 3–5 animals per group. Results are expressed as means. Student's t-test was used for statistical analysis. n.s. stands for not significant, p>0.05.

To determine the contribution of other myeloid or lymphoid cell populations to the IFN-β production after *L. monocytogenes* infection, we employed tissue specific reporter mice since these mice allow tissue specific replacement of the IFN-β gene by the luciferase reporter in the IFN-β^+/floxβ-luc^ mice [30]. These mice were crossed with mice expressing Cre recombinase under various tissue specific promoters. CD19cre mice were used to implement the reporter activity in B cells, CD4cre mice in T cells and LysMcre mice in monocytes/macrophages and granulocytes. All reporter mice were heterozygous for both markers to allow normal cellular development as well as potential production of IFN-β in all cells. Comparison of such tissue specific reporter mice after Listeria infection with the “global” IFN-β reporter mouse (IFN-β^+/Δβ-luc^) by whole body *in vivo* imaging demonstrated that T- and B-cells did not have any apparent impact on the IFN-β production after infection by *L. monocytogenes* (Fig. 5A).

Moreover, about 80% of pDCs are supposed to exhibit recombination in CD4cre mice [37]. Nevertheless, these mice did not show any luciferase production, confirming our data from above. Thus, we conclude that pDCs do not contribute to the type I IFN production during *Listeria monocytogenes* infection. Only cells, in which the LysM-promoter is active, significantly contribute to the IFN-β signal in spleen at 24 and in lymph nodes at 48 hours p.i. (Fig. 5A). Of note, the bioluminescence signal in such mice is lower than in the mice displayed in Fig. 1A. Obviously, the signal is quenched by the black skin and fur of the recombinant C57BL/6 mice.

To overcome the restrictions of *in vivo* imaging with black mice, the results were corroborated by *ex vivo* quantification of luciferase activity (Fig. 5B). Using this assay, we could confirm that T- and B-cells did not contribute to luciferase activity in spleen and lymph nodes at both time points. In contrast, LysMcre specific IFN-β reporter mice contributed with similar levels of luciferase activity compared to IFN-β^+/Δβ-luc^ mice. Hence, LysM-expressing cells almost exclusively produce IFN-β in both spleen and lymph nodes upon infection with *L. monocytogenes*.

### Confirming the role of LysM-expressing cells during *Listeria monocytogenes* infection

**Figure 6:**
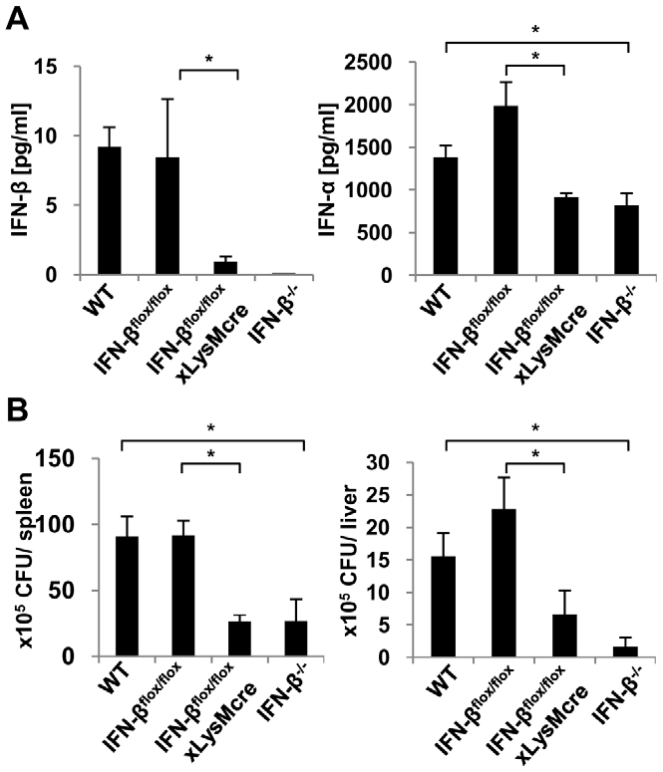
Ablation of IFN-β production in LysM-expressing cells is equal to the overall absence of IFN-β. Mice of indicated genotypes were infected intravenously with 5×10^5^ *L. monocytogenes* LO28. 24 hours post infection mice were sacrificed and spleens, livers and serum were isolated. “WT” stands for C57BL/6, “IFN-β^flox/flox^” stands for IFN-β^floxβ-luc/floxβ-luc^ and “IFN-β^flox/flox^xLysMcre” stands for IFN-β^floxβ-luc/floxβ-luc^ x LysMcre. **A**) Serum levels of IFN-β and total IFN-α were analyzed by ELISA. **B**) Bacterial numbers from spleens and liver were calculated and presented as colony forming units (CFUs).Graphs are taken from 1 representative experiment with 3–5 mice per group. The experiment was repeated twice. Student's t-test was used for statistical analysis. *p<0.05.

To confirm the contribution of LysM positive cells to the type I IFN response against *L. monocytogenes* as well as to characterize their role during infection with this pathogen, we analyzed homozygous mice, in which the gene encoding IFN-β was cell-specifically deleted on both alleles. As a control, we included IFN-β^floxβ-luc/floxβ-luc^ mice to demonstrate that the genetic changes in the targeted locus have no impact on their own on the course of infection. C57BL/6, IFN-β^floxβ-luc/floxβ-luc^, IFN-β^floxβ-luc/floxβ-luc^ x LysMcre and IFN-β^−/−^ mice were infected with *Listeria monocytogenes* and 24 hours p.i. serum levels of type I IFN were determined by ELISA (Fig. 6A). Importantly, IFN-β production was identical in C57BL/6 and IFN-β^floxβ-luc/floxβ-luc^ mice, while the extent of IFN-β expression was dramatically reduced in IFN-β^floxβ-luc/floxβ-luc^ x LysMcre mice. As a consequence, since type I IFN induction in macrophages depends on feedback signaling, total IFN-α levels were also markedly reduced in IFN-β^floxβ-luc/floxβ-luc^ x LysMcre mice and they were similar to the levels in IFN-β^−/−^ mice (Fig. 6A).

Consistent with these results, the CFUs isolated from spleen and liver of C57BL/6 and IFN-β^floxβ-luc/floxβ-luc^ mice did not differ significantly, while IFN-β^floxβ-luc/floxβ-luc^ x LysMcre and IFN-β^−/−^ mice showed strongly reduced bacterial numbers in the organs 24 hours after infection (Fig. 6B). Moreover, comparison of IFN-β^floxβ-luc/floxβ-luc^ mice with IFN-β^floxβ-luc/floxβ-luc^ x CD11ccre, where IFN-β production is ablated in dendritic cells, showed no significant differences in bacterial loads in spleen and liver, as well as in serum levels of type I IFN (Figure S2).

Taken together, our results showed that preventing LysM-expressing cells from IFN-β production is sufficient to avoid the detrimental effects of type I IFN during *L. monocytogenes* infection. Thus, such cells are apparently the major source of IFN-β during murine listeriosis.

### Neutrophils do not produce IFN-β

**Figure 7:**
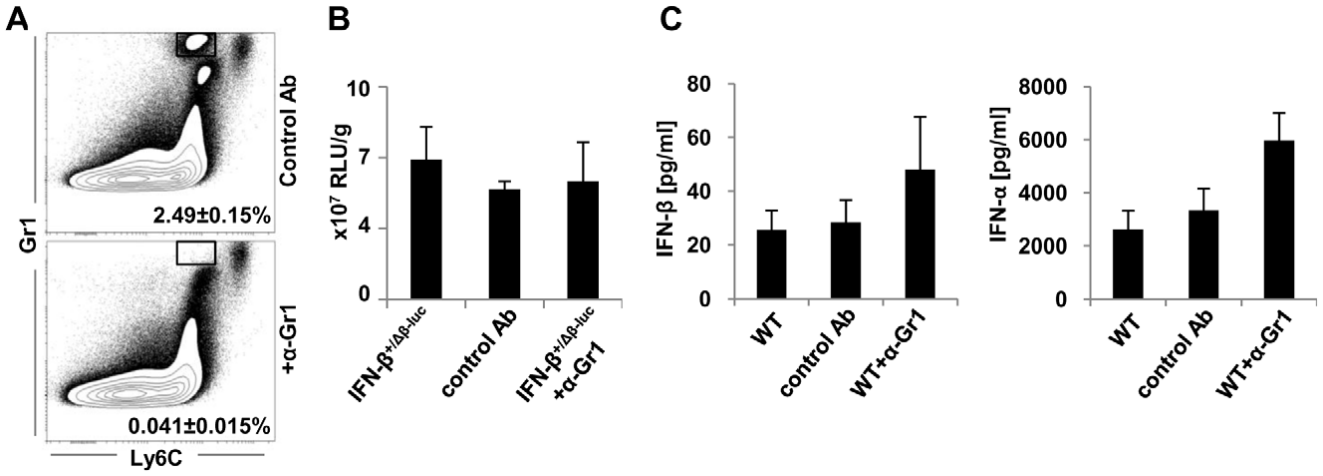
Depletion of granulocytes does not influence the amount of type I IFN. Granulocytes were depleted 24 hours prior to infection of mice with 5×10^5^ *L. monocytogenes* LO28. As isotype control, rat IgG was used. 24 hours post infection IFN-β^+/Δβ-luc^ and C57BL/6 mice were sacrificed, spleens and serum were harvested. **A**) Percentage of granulocytes in spleens. **B**) For quantification of luciferase activity, spleen homogenates from IFN-β^+/Δβ-luc^ mice were analyzed in a luminometer. **C**) Serum levels of IFN-β and total IFN-α from C57BL/6 mice were analyzed by ELISA. “+α-Gr1” stands for mice depleted of granulocytes. “WT” stands for C57BL/6 mice. Graphs are taken from 1 representative experiment with 3–5 mice per group. The experiment was repeated 3 times.

The LysM gene in mice is active mostly in monocyte/macrophages and neutrophils [38], [39]. Neutrophils are essential during the early innate response to *Listeria monocytogenes* and are known to be capable to produce various cytokines, such as IL-1β, IL-6, TNF-α, IL-12, MIP-1α, MIP-1β, IL-18 and reactive oxygen intermediates in response to different stimuli [40]–[43]. However, the contribution of neutrophils to type I IFN production is still unclear. Therefore, we wanted to determine whether neutrophils are involved in IFN-β production after *L. monocytogenes* infection. To answer this question, we depleted granulocytes 24 hours prior infection after carefully titrating the antibody. Depletion of neutrophils was close to complete (Fig. 7A), while changes in other cell populations were negligible (Figure S3). *Ex vivo* analysis of luciferase activity in IFN-β^+/Δβ-luc^ mice depleted of neutrophils revealed, that these cells did not have a significant impact on luciferase expression (Fig. 7B). In addition, similar amounts of IFN-β and total IFN-α in serum were found in C57BL/6 mice despite of the neutrophil depletion (Fig. 7C). Hence, LysM-positive monocyte/macrophages are the population that is responsible to almost entirely produce type I IFN after *Listeria monocytogenes* infection.

### Tip-DCs express LysM and are the main source of IFN-β after *Listeria monocytogenes* infection

**Figure 8:**
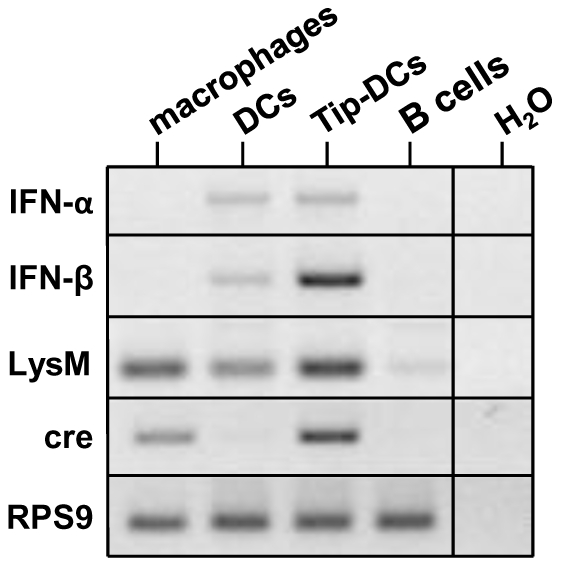
LysM-expressing cells include Tip-DCs - the main source of IFN-β produced after *Listeria monocytogenes* infection. IFN-β^+/floxβ-luc^ x LysMcre mice (n = 5) were infected intravenously with 5×10^5^ *L. monocytogenes* LO28 strain. 24 hours after infection mice were sacrificed, splenic cells were isolated and subjected to FACS sorting. RNA of the isolated cells was extracted, reverse-transcribed and subjected to PCR for the indicated genes. RPS9 was used as housekeeping gene to control the amount of cDNA employed in the assay. As a negative control H_2_O was used instead of cDNA template.

It is known, that the LysM promoter is active not only in monocyte/macrophages and neutrophils, but also in part of the CD11c^+^ DCs, while no expression is observed in T and B cells [44]. Since it was recently shown that CD11b^+^CD11c^int^Ly6C^+^ cells previously defined as Tip-DCs are the major IFN-β producers upon Listeria infection [29], we wanted to know whether Tip-DCs belong to LysM-expressing monocyte /macrophage population and whether we could confirm the production of IFN-β by such cells. Therefore, we sorted CD11b^+^CD11c^−^macrophages, CD11c^+^CD11b^−^ dendritic cells (DCs), CD11b^+^CD11c^int^Ly6C^+^ Tip-DCs and B220^+^CD11c^−^ B cells as a negative control from IFN-β^+/floxβ-luc^ x LysMcre mice 24 hours after Listeria infection and tested for LysM, LysMcre as well as IFN-β expression by RT-PCR. A strong signal was observed for LysM in Tip-DCs and macrophages and, to a lesser extent, in dendritic cells (Fig. 8). Cre recombinase was expressed in macrophages and in Tip-DCs, but not in DCs. IFN-β mRNA was highly pronounced in Tip-DCs and weakly in DCs. Faint bands were also detectible for IFN-α in these cells. In contrast, CD11b^+^CD11c^−^ macrophages failed to produce any detectable amount of IFN-β or IFN-α. Taking together, our results indicate that cells defined as Tip-DCs are within LysM-expressing cells and are responsible for the major IFN-β production upon *Listeria monocytogenes* infection.

## Discussion

Type I IFN are extremely pleiotropic cytokines. For instance, they are involved in defense against many viruses [45]–[47], could be either protective or detrimental during non-viral pathogens [7], [8], act in cancer surveillance [48], [49], but they are also effector molecules in toxic shock elicited by LPS [50] and TNF [51]. In addition, recombinant type I IFN is used in the clinics as therapy against certain cancers as well as for treatment of chronic infections caused by HBV [52], HCV [53] and multiple sclerosis [54]. Thus, these molecules could be of great health benefit but could also elicit serious detrimental effects. Therefore, knowledge about the cells involved in production of type I IFN and their regulation is of utmost importance for the understanding of the IFN system.

*Listeria monocytogenes* is an intracellular pathogen, broadly used as a model to investigate host-pathogen interactions. Different groups have shown the production of type I IFN *in vitro* and *in vivo* upon infection with *Listeria monocytogenes* [10], [11]. We followed IFN-β induction in the mouse using non-invasive whole body *in vivo* imaging. This way we derived a spatiotemporal resolved picture of the early IFN-β response during Listeria infection. We focused on production of IFN-β, because IFN-β appears to be the primary member of type I IFN family that is induced after infection with Listeria [16], [17] and initiates the cascade of type I IFN in most cell types [4], [5].

By a genetic approach we investigated the cells that are responsible for IFN-β production during murine listeriosis. We could show that IFN-β is almost exclusively produced by LysM-expressing cells in the spleen as well as in colonized lymph nodes. LysM promoter is highly expressed in monocytes/macrophages and neutrophils [44]. However, the use of the LysMcre mouse does not allow distinction between myeloid cell populations, although lower expression in CD11c^+^ cells was reported [55].

Macrophages have been identified as one of the target cell population in the murine spleen [26]. Besides, macrophages play a major role in the early innate defense against *Listeria monocytogenes* via secretion of different cytokines [20], [56]. However, DCs were also observed to be directly infected by *L. monocytogenes* [57]. Since IFN induction requires intracellular Listeria, we would have expected that DCs, in addition to monocytes/macrophages, would be involved in IFN-β production. This is apparently not the case, because i) only negligible amounts of IFN-β are produced in infected mice, in which IFN-β is conditionally deleted by LysMcre. This also excludes a major contribution of a non-lymphoid tissue associated cell. ii) Similar amounts of reporter luciferase are produced in the global reporter mice and in the mice reporting production of IFN-β by monocytes/macrophages and neutrophilic granulocytes. Although LysMcre is supposed to be expressed by a small population of DCs [44], CD11ccre mice experiments confirm that conventional DCs do not significantly contribute to IFN-β production. We also could exclude the contribution of pDC. The depletion of this cell population had no effect on the overall production of type I IFN. This extends the *in vitro* data of Stockinger et al. [28] to the *in vivo* situation. Finally, the involvement of neutrophils that are expressing LysMcre was also excluded by a depletion experiment.

Stockinger et al. [28] defined cells with surface antigens characteristic of macrophages as major producers of type I IFN during Listeria infection. According to the recent publication of Dresing et al. [29], TNF-α and iNOS producing Tip-DCs, that share markers with macrophages, were the main cellular source of IFN-β in the course of Listeria infection. Our data showed that a LysM-expressing cell population is responsible for IFN-β production in murine listeriosis. Testing sorted macrophages, DCs and Tip-DCs for type I IFN and LysM expression revealed that the LysM promoter was active in all three cell populations. However, only Tip-DCs were able to express vast amounts of IFN-β in agreement with Dresing et al. [29]. Interestingly, such Tip-DCs showed strong expression of LysM. This sheds some doubts on the DC nature of Tip-DCs, which was originally defined by their T cell priming capacity in mixed lymphocyte reaction [29]. Thus, Tip-DCs might as well be defined as inflammatory monocytes.

DCs might be involved only at the very early time points after infection and IFN-β production might be below our detection limit. We think this is unlikely given the extreme sensitivity of our reporter system. Alternatively, infected DCs might undergo apoptosis before they are able to significantly contribute to IFN production or IFN production in DCs might be inhibited by *L. monocytogenes*. The latter notion would be in agreement with the fact that pDCs also do not produce IFN-β.

A similar argument we would use for the absence of IFN-β production by hepatocytes. Such cells are known to be severely infected by *L. monocytogenes* during the complete course of infection. Nevertheless, no significant signal was observed in the reporter mice from liver. The absence of a contribution of Kupffer cells was expected since such cells are known to only bind Listeria but are not directly infected [58]. IFN-β induction requires cytosolic residence of Listeria.

The colonization of lymph nodes after i.v. infection with *L. monocytogenes* has been ignored so far, although it is known that such bacteria are able to infect lymph nodes after subcutaneous [59] or oral application [36]. Here we have shown that *Listeria monocytogenes* successfully colonizes cervical and inguinal lymph nodes also after i.v. infection. We noticed lymph node colonization due to the activation of the IFN-β reporter in such tissues. This impressively demonstrates the importance of using reporter systems that allow holistic unbiased observations.

Colonization of lymph nodes could have taken place via dissemination of bacteria from the “classical” target organs spleen and liver. The delayed production of IFN-β in such organs would be in agreement with this notion. However, production of IFN-β correlated with the number of bacteria found in the colonized lymph nodes, which also showed a delayed increase. Thus, lymph nodes obviously are also primary target organs for *L. monocytogenes*, although they do not show the same penetrance as spleen and liver.

Together, our work compellingly shows that LysM-expressing Tip-DCs are the major producers of IFN-β during murine listeriosis, which is responsible for the induction of the type I IFN cascade in most cell types. The expression of LysM sheds some doubt on the DC nature of such cells. We also show *in vivo* that pDCs, the cell type that is able to produce type I IFN independent of IFN-β and neutrophils do not contribute at all to the overall production of type I IFN during murine listeriosis. Having settled this, the question now arises: why cDCs, that are infected at least early after i.v. application or hepatocytes that are known to be heavily infected by *L. monocytogenes*, do not produce type I IFN.

## Materials and Methods

### Ethics statement

Mouse care and experimental procedures were performed under the approval of local authority Niedersächsisches Landesamt für Verbraucherschutz und Lebensmittelsicherheit (LAVES). Permit numbers for this study are 33.11.42502-04-067/07 and 33.42502-071/06.

### Mice

All mice were bred at the animal facility of the Helmholtz Centre for Infection Research (HZI) and maintained under specific pathogen-free conditions. For experiments, female mice 8 to 12 weeks of age were used. All mice used in this study were on the C57BL/6 background. IFN-β^−/−^ [60] mice were backcrossed onto C57BL/6 for more than 15 generations. Conditional deletion/reporter mice IFN-β^floxβ-luc^ were generated using C57BL/6 ES-cells (Bruce4). To replace the IFN-β CDS by luciferase in germ line (IFN-β^Δβ-luc^), IFN-β^floxβ-luc^ mice were crossed with K14cre mice [61]. To receive tissue specific IFN-β deletion as well as reporter expression, IFN-β^floxβ-luc^ were crossed to CD19cre [62], CD4cre [63] and LysMcre [44] mice respectively (kindly provided by Dr. Angela Schippers, HZI). CD11ccre mice [64] were kindly provided by Prof. Dr. Ulrich Kalinke, Twincore. Additionally IFN-β^+/Δβ-luc^ mice were crossed with albino C57BL/6 (C57BL/6-Tyr<c-2J>) kindly provided by Thomas Blankenstein (MDC, Berlin) to improve *in vivo* imaging.

### Splenocyte isolation

Spleen cells were prepared by gently flushing the spleen with IMDM supplemented with antibiotics (100 U/ml penicillin and 100 µg/ml streptomycin), 10% FCS, 50 µM β-ME, and 2 mM L-glutamine. Erythrocytes were lysed for 2 min in ACK buffer (0.15 M NH_4_Cl, 10 mM KHCO_3_, and 0.1 mM EDTA) and cells were washed two times with PBS. Cell clumps were removed by passaging through a 50 µm nylon filter. Preparation was conducted strictly at 0°C.

### Antibodies

Single cell suspensions were treated with anti-mouse CD16/CD32 BD Fc Block (2.4G2, Becton Dickinson, NJ, USA) for 10 min followed by staining with appropriate mAbs for 20 minutes on ice. Abs used in this work included PE anti-mPDCA-1clone JF05-1C2.4.1 (Miltenyi Biotec), APC CD11c clone N418 (eBioscience, San Diego, USA), Pacific Blue CD11b clone M1/70.15 (Invitrogen), APC-Cy7 B220 clone RA3-6B2 (BD Pharmingen, New York, USA), anti-mouse Ly6G PE-Cy7 (Gr1, eBioscience, San Diego, USA), biotynylated anti-mouse Ly6C (Pharmingen, New York, USA), streptavidin APC-Cy7 (Pharmingen, New York, USA). Flow cytometric analysis and sorting was performed using LSRII (Becton Dickinson, NJ, USA. The data were analyzed using FACSDiva (Becton Dickinson) software.

### RT-PCR

Total RNA was extracted from spleen cells using RNeasy mini kit (Qiagen) according to the manufacturer's instructions. DNA contamination in the total RNA preparation was eliminated using DNase I (Qiagen). RevertAid First Strand cDNA Synthesis Kit (Fermentas) with oligo(dT) primers was used for reverse transcription of purified RNA. PCR was performed using the Promega kit according to the instructions of the supplier. The following primers were used for RT-PCR: 5′-CATCAACTATAAGCAGCTCCA-3′, 5′-TTCAAGTGGAGAGCAGTTGAG-3′ for IFN-β; 5′-ATGGCTAGACTCTGTGCTTTCCT-3′, 5′-AGGGCTCTCCAGATTTCTGCTCTG-3′ for panIFN-α [28]; 5′-AACCCCAAGAGCTGTGAATG-3′, 5′-TCGGTTTTGACAGTGTGCTC-3′ for LysM; 5′-ACG ACC AAG TGA CAG CAA TG-3′, 5′- CTC GAC CAG TTT AGT TAC CC-3′ for cre recombinase; 5′-CTGGACGAGGGCAAGATGAAGC-3′, 5′-TGACGTTGGCGGATGAGCACA-3′ for ribosomal protein S9 (RPS9).

### Bacteria

*Listeria monocytogenes* strain LO28 serotype 1/2c [65] (kindly provided by Thomas Decker, Vienna, Austria) and EGDe serotype 1/2a [66] was grown in Brain Heart Infusion (BHI) infusion or on BHI-plates (Difco, Detroit, MI) at 37°C overnight. The next day, suspensions were diluted and grown until reaching log-phase. Bacteria were then centrifuged, washed several times and resuspended in sterile PBS. Concentrations of bacteria were determined by measurement at OD_600_ and confirmed by plating serial dilutions on appropriate agar plates.

### Detection of luciferase

For the determination of the enzymatic activity of luciferase, cells were lysed in Reporter Lysis Buffer (Promega). For luciferase activity assays from tissue, weight of tissue fragments was determined and fragments were homogenized in proportional volumes of Reporter Lysis Buffer using Lysing Matrix A on a FastPrep-24 (MP Biomedicals). Lysates were mixed with LARII (Promega) and measured in a luminometer (Berthold). For *in vivo* imaging, mice were injected i.v. with 150 mg/kg of D-luciferin (Synchem) in PBS, anesthetized using Isofluran (Baxter) and monitored using an IVIS 200 imaging system (CaliperLS). Photon flux was quantified using the Living Image 3.2 software (CaliperLS).

### ELISA

Sera of infected mice were collected and levels of IFN-β and total IFN-α were measured by ELISA according to manufacturer's protocols (PBL InterferonSource).

### Infections

Mice were infected intravenously (i.v.) with *Listeria monocytogenes* LO28 or EGDe strains. 5×10^5^ CFU/mouse is lethal dose (25×LD_50_), mice start dying at day 2–3 after infection. 2×10^3^ CFU/mouse is a sublethal dose (0.1×LD_50_), all mice are able to clear the bacterial around day 7 after infection. For determination of bacterial loads, spleens, livers and lymph nodes of sacrificed mice were removed and homogenized in 1 ml PBS supplemented with 0.2% NP-40. Serial dilutions of homogenates were plated on BHI agar plates and colonies were counted after overnight incubation at 37°C.

### Depletion experiments

Plasmacytoid dendritic cells and granulocytes were depleted *in vivo* by intravenous injection of 100 µg anti-mPDCA-1 mAb (clone JF05-1C2.4.1, functional grade; Miltenyi Biotec) or 10 µg anti-Gr1 mAb (clone RB6-8C5) 24 hours prior *Listeria monocytogenes* infection. As an isotype control, rat IgG (Jackson ImmunoResearch Laboratories, Inc.) was used. Successful depletion of pDCs and granulocytes was determined by FACS analysis 24 hours after infection.

## Supporting Information

Figure S1 **Overview of transgenic mice used in this work.** The generation of the global IFN-β reporter mouse has been previously described [30] and is since then maintained independent of cre expression. In brief, the targeted locus contains a luciferase gene (dark blue arrow) with a preceding polyA signal (black box) to avoid unspecific reporter activity. Two loxP sites (black arrowheads) allow cre-dependent replacement of the *ifnb* coding sequence by the luciferase reporter (light blue arrow) which is then driven by the endogenous *ifnb* promoter. In all reporter mice we keep one wt *ifnb* allel to allow IFN-β expression. Tissue specific reporter mice were obtained from breeding IFN-β^floxβ-luc/floxβ-luc^ mice with mice expressing cre (red arrow) in a cell type specific manner. Cre activity (light red) in the given cell population (within the red circle) then allows *ifnb* promoter dependent reporter activity while the reporter cannot be activated in cells without cre expression (dark red). Tissue specific knockout mice carry two cre dependent alleles. Therefore in cells expressing cre both *ifnb* coding sequences are deleted. However, in cells without cre expression the IFN-β production is normal despite the genomic alterations introduced into the locus.
(TIF)

Figure S2 **Disruption of IFN-β production in CD11c+ dendritic cells does not alter type I IFN production.** Mice of indicated phenotypes were infected intravenously with 5×10^5^ *L. monocytogenes* LO28. 24 hours post infection mice were sacrificed, spleens, livers and serum were isolated. **A**) Bacterial numbers from spleens and livers were calculated and presented as colony forming units (CFUs). **B**) Serum levels of IFN-β and total IFN-α were analyzed by ELISA. IFN-β^flox/flox^ stands for IFN-β^floxβ-luc/floxβ-luc^, IFN-β^flox/flox^xCD11ccre stands for IFN-β^floxβ-luc/floxβ-luc^ x CD11ccre. Graphs are taken from 1 representative experiment with 5 mice per group. The experiment was repeated 3 times. Student's t-test was used for statistical analysis. n.s. stands for not significant, p>0.05.
(TIF)

Figure S3 **Depletion of granulocytes does not influence other cell populations.** Granulocytes were depleted 24 hours prior to infection of mice with 5×10^5^ *L. monocytogenes* LO28. As isotype control, rat IgG was used. 24 hours post infection C57BL/6 mice were sacrificed, spleens were isolated and depletion was controlled by testing spleen samples. **A**) Gating strategy of splenic Tip-DCs. **B**) Analysis of depleted populations. Populations other than granulocytes are not significantly influenced by anti-Gr1 antibody mediated depletion.
(TIF)
