# Supplementary figures and images for: Production of IFN-β during Listeria monocytogenes Infection Is Restricted to Monocyte/Macrophage Lineage
Source: PLoS One. 2011 Apr 11;6(4):e18543. doi: 10.1371/journal.pone.0018543 (PMC3073975; doi:10.1371/journal.pone.0018543)

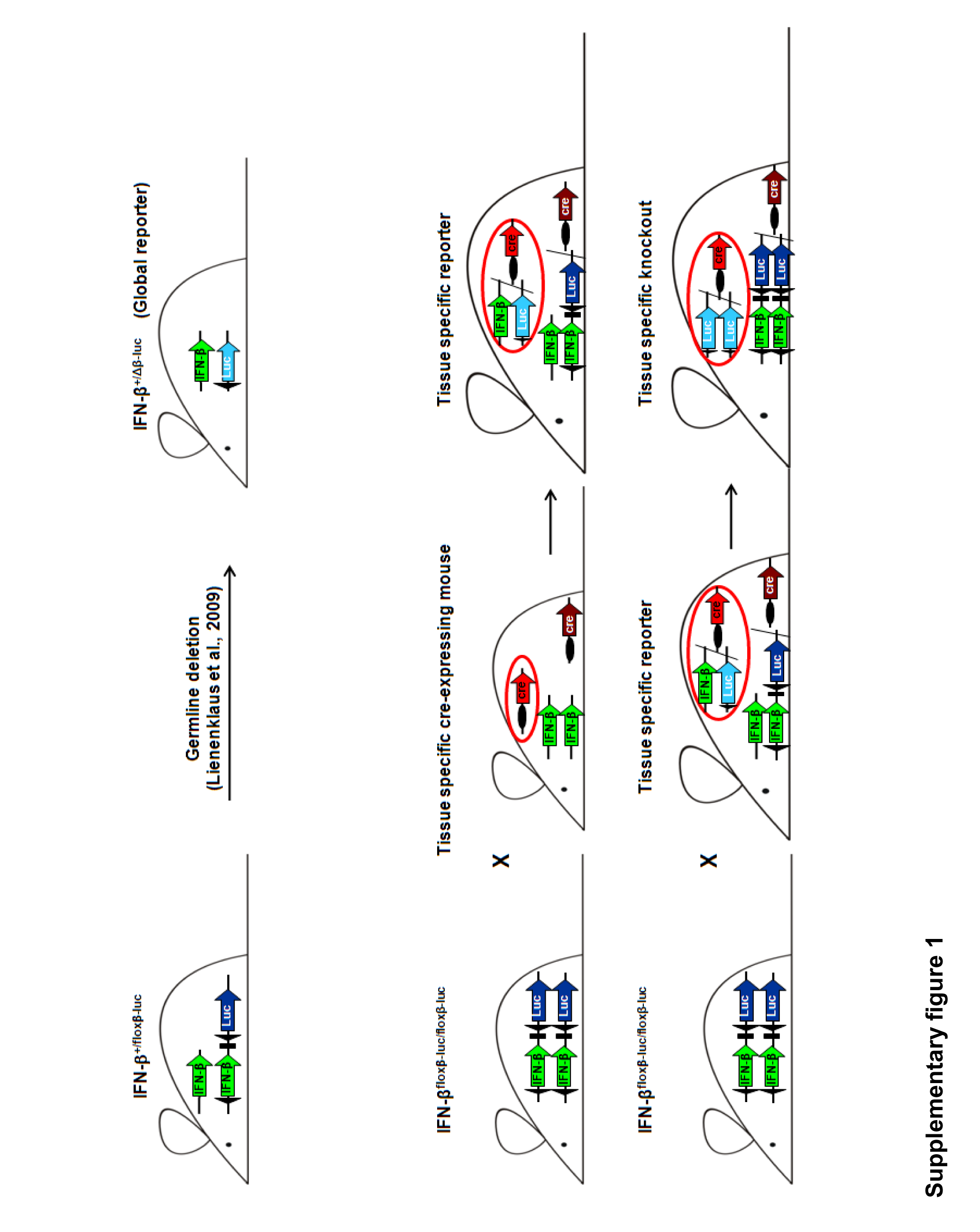

Supplement: Figure S1 — Overview of transgenic mice used in this work. The generation of the global IFN-β reporter mouse has been previously described [30] and is since then maintained independent of cre expression. In brief, the targeted locus contains a luciferase gene (dark blue arrow) with a preceding polyA signal (black box) to avoid unspecific reporter activity. Two loxP sites (black arrowheads) allow cre-dependent replacement of the ifnb coding sequence by the luciferase reporter (light blue arrow) which is then driven by the endogenous ifnb promoter. In all reporter mice we keep one wt ifnb allel to allow IFN-β expression. Tissue specific reporter mice were obtained from breeding IFN-βfloxβ-luc/floxβ-luc mice with mice expressing cre (red arrow) in a cell type specific manner. Cre activity (light red) in the given cell population (within the red circle) then allows ifnb promoter dependent reporter activity while the reporter cannot be activated in cells without cre expression (dark red). Tissue specific knockout mice carry two cre dependent alleles. Therefore in cells expressing cre both ifnb coding sequences are deleted. However, in cells without cre expression the IFN-β production is normal despite the genomic alterations introduced into the locus. (TIF) [file pone.0018543.s001.tif]

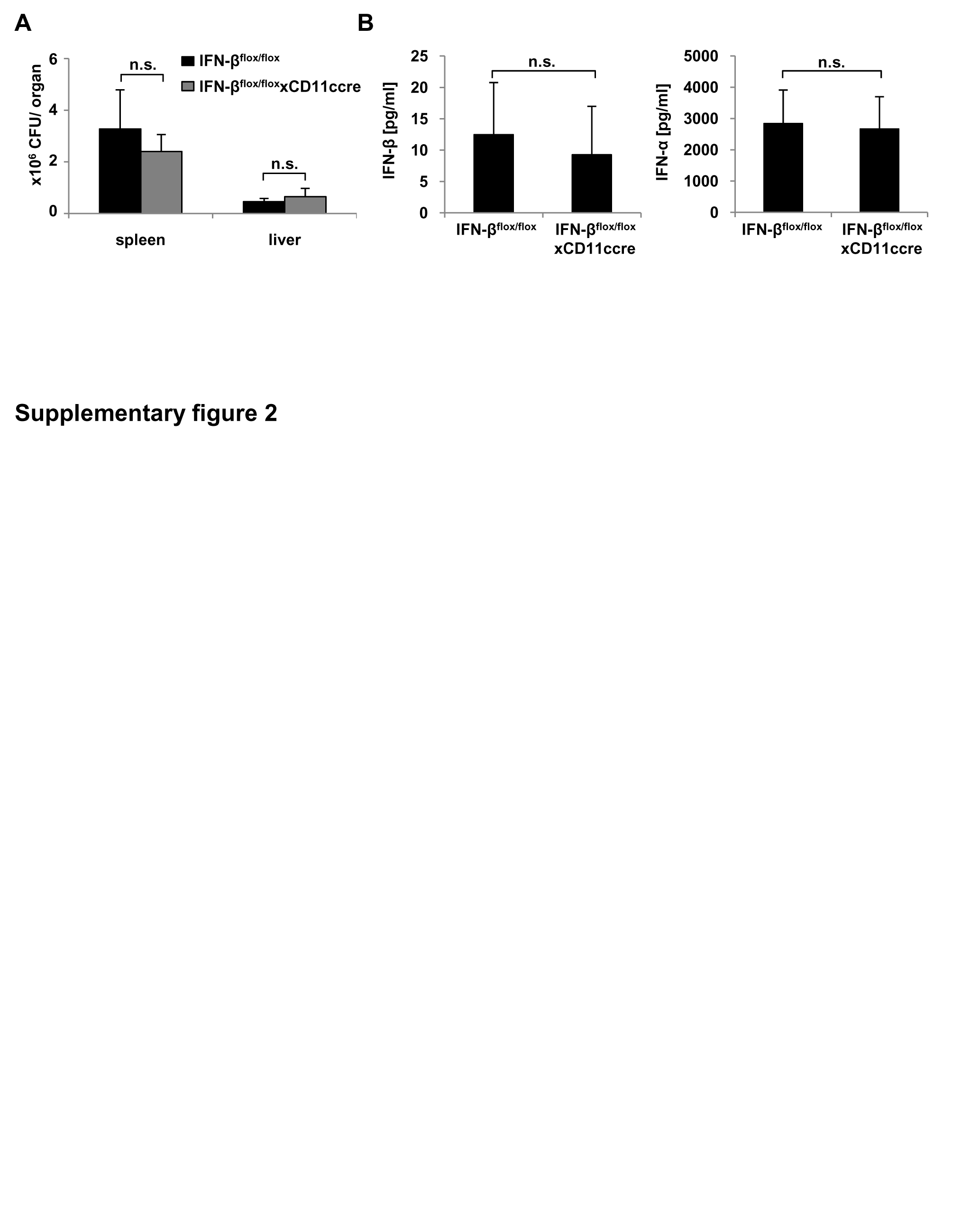

Supplement: Figure S2 — Disruption of IFN-β production in CD11c+ dendritic cells does not alter type I IFN production. Mice of indicated phenotypes were infected intravenously with 5×10^5^ L. monocytogenes LO28. 24 hours post infection mice were sacrificed, spleens, livers and serum were isolated. A) Bacterial numbers from spleens and livers were calculated and presented as colony forming units (CFUs). B) Serum levels of IFN-β and total IFN-α were analyzed by ELISA. IFN-βflox/flox stands for IFN-βfloxβ-luc/floxβ-luc, IFN-βflox/floxxCD11ccre stands for IFN-βfloxβ-luc/floxβ-luc x CD11ccre. Graphs are taken from 1 representative experiment with 5 mice per group. The experiment was repeated 3 times. Student's t-test was used for statistical analysis. n.s. stands for not significant, p>0.05. (TIF) [file pone.0018543.s002.tif]

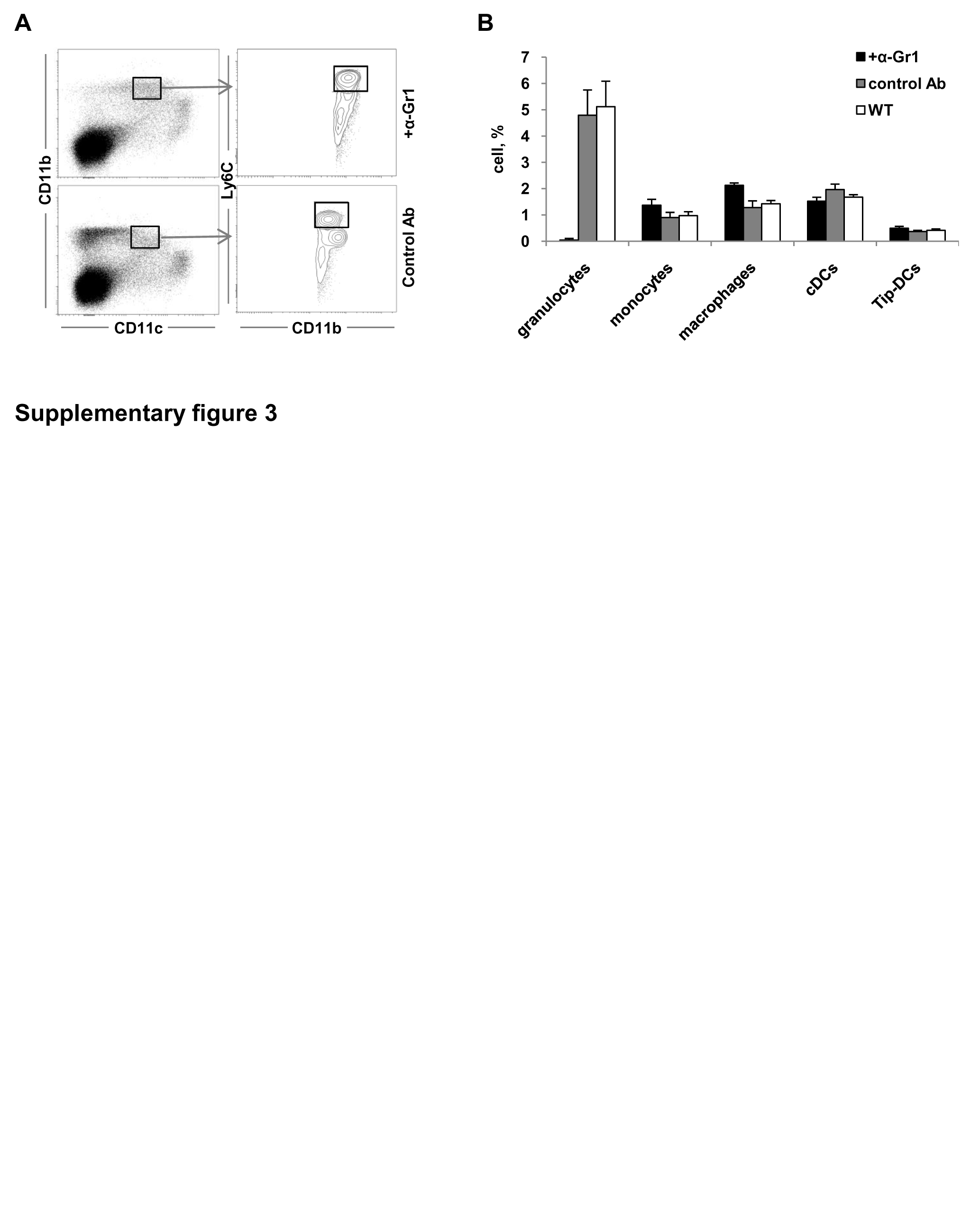

Supplement: Figure S3 — Depletion of granulocytes does not influence other cell populations. Granulocytes were depleted 24 hours prior to infection of mice with 5×10^5^ L. monocytogenes LO28. As isotype control, rat IgG was used. 24 hours post infection C57BL/6 mice were sacrificed, spleens were isolated and depletion was controlled by testing spleen samples. A) Gating strategy of splenic Tip-DCs. B) Analysis of depleted populations. Populations other than granulocytes are not significantly influenced by anti-Gr1 antibody mediated depletion. (TIF) [file pone.0018543.s003.tif]
